# Gendermetrics.NET: a novel software for analyzing the gender representation in scientific authoring

**DOI:** 10.1186/s12995-016-0133-6

**Published:** 2016-09-15

**Authors:** Michael H. K. Bendels, Dörthe Brüggmann, Norman Schöffel, David A. Groneberg

**Affiliations:** 1 Division of Computational Medicine, The Institute of Occupational Medicine, Social Medicine and Environmental Medicine, Goethe-University, Frankfurt, Germany; 2 Division of Female Health and Prevention, The Institute of Occupational Medicine, Social Medicine and Environmental Medicine, Goethe-University, Frankfurt, Germany; 3 Division of Clinical Occupational Medicine, The Institute of Occupational Medicine, Social Medicine and Environmental Medicine, Goethe-University, Frankfurt, Germany

**Keywords:** Gender, Gender analysis, Scientometric analysis, Bibliometric analysis, Software tool, Science structure

## Abstract

**Background:**

Imbalances in female career promotion are believed to be strong in the field of academic science. A primary parameter to analyze gender inequalities is the gender authoring in scientific publications. Since the presently available data on gender distribution is largely limited to underpowered studies, we here develop a new approach to analyze authors’ genders in large bibliometric databases.

**Results:**

A SQL-Server based multiuser software suite was developed that serves as an integrative tool for analyzing bibliometric data with a special emphasis on gender and topographical analysis. The presented system allows seamless integration, inspection, modification, evaluation and visualization of bibliometric data. By providing an adaptive and almost fully automatic integration and analysis process, the inter-individual variability of analysis is kept at a low level. Depending on the scientific question, the system enables the user to perform a scientometric analysis including its visualization within a short period of time.

**Conclusion:**

In summary, a new software suite for analyzing gender representations in scientific articles was established. The system is suitable for the comparative analysis of scientific structures on the level of continents, countries, cities, city regions, institutions, research fields and journals.

## Background

In the media and in scientific discussion, it is not only debated that women earn less payment in equal positions, but also less frequently climb the stairs to high ranked academic positions, e.g. in the field of medicine [8, 15, 19]. This discussion is not only limited to academic medicine, but to almost every area of science, as demonstrated by news, comments, statements or studies and reviews in renowned scientific journals such as the Science, Nature, The New England Journal of Medicine or The Lancet [3, 5, 11, 16]. A special indicator of gender representation is the position of an author in the list of authors with the first authorships and the last, alternatively senior authorships being the most prominent positions; numerous studies have focused on this issue [1, 2, 7]. However, despite the availability of a variety of different sophisticated bibliometric software tools [9, 14, 17, 18], there exists to our knowledge up to now no integrative tool for the fast and large scale analysis of gender distributions in scientific articles. Instead, gender-related analysis is still done by employing cascaded single systems performing separately the different processing steps like integration of raw data, location assignment, sex determination of the authors and statistical analysis including topic related aspects, collaboration analysis and the computation of bibliometric indices, often combined with extensive manual processing steps introducing an undesirable inter-individual variability into the analysis [7]. To close this gap we have developed the software suite Gendermetrics.NET which serves as an integrative tool for analyzing bibliometric data with a special emphasis on gender representation and the incorporation of the article-related geo-coordinates allowing for various topographical analyses. A special design criterion is the combination of both, offline and online services for integrating raw bibliometric data and the authors’ gender. In the following, we present design and key functionality of the software. Subsequently, selected core routines of the software are demonstrated. Finally, to exemplify the working process, a standard procedure of evaluating bibliometric data is outlined. This article serves as a brief guide on how to successfully design and implement a multiuser bilbiometric software tool.

## Methods

### Software design

**Fig. 1:**
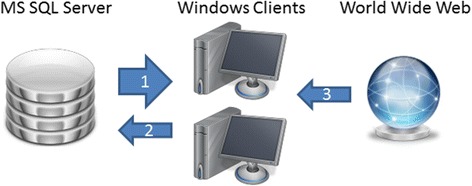
Software-Architecture. All global (non-project-specific) data and its associated procedures for both integration of raw bibliometric data and algorithmic gender analysis are managed centrally in a Microsoft-SQL-Server database (*left*). The data is provided to downstream Windows clients (*middle*) hosted within the LAN (*arrow 1*). These remote Windows clients – representing the working environment for the scientists - include all project-specific data like publications, authors, subject areas, institutions, cities and countries, related bibliometric indices, the statistical evaluation and figure plots. Additionally, the system allows the user to make client-side updates to the database in the sense of a global learning function improving subsequent integration/identification processes (*arrow 2*). To complement this database supported offline integration yet unresolved/unidentified items are evaluable by applying the integrated online web services (*right*) like BingMapSearch, GoogleMaps and Google (*arrow 3*). The chosen software architecture ensures the implementation of a simultaneous multi-user system

All global (non-project-specific) data and its associated procedures for both integration of raw bibliometric data and algorithmic gender analysis are managed centrally in a Microsoft SQL-Server database (Fig. 1). The database includes essentially the names of all countries in the world and their cities with geo-coordinates (approximately 350,000 cities), a gender table for the lexical gender determination by forenames (approximately 73,000 forenames) and an expandable dictionary for the context-sensitive substitution of text modules. The latter is used to customize the data integration and the normalization process in the sense of a learning function.

The data is provided to downstream windows clients hosted within the local area network (LAN). These remote Windows clients – representing the working environment for the scientists – include all project-specific data like publications, authors, subject areas, institutions, cities and countries, related bibliometric indices, the statistical evaluation and figure plots. All project data is stored in one file including the statistical evaluation, the figures are redrawn. This allows easy sharing and caching of project data. The intrinsically compressed file has a typical size of about 250–500 bytes per article. Conceptually, the system allows the user to make client-side updates to the database in the sense of a global learning function improving subsequent integration and identification processes for all users. The consistent application of the learning function allows the complete algorithmic reintegration of formerly integrated data. To complement this database supported offline integration yet unresolved items are evaluable by generating automated queries to the integrated online web services like BingMapSearch, GoogleMaps and Google. The software architecture is a simultaneous multi-user system, i.e. a system in which users can connect and make changes to the same database.

The Windows client was written in C# using the Microsoft Visual Studio 2015 (Microsoft, Redmond, USA) extended by the powerful DevExpress WinForms-Extension (DevExpress, Glendale, California, USA). The latter improves and simplifies both inspection and visualization of bibliometric and geographic data, e.g. by providing a powerful and flexible map and chart control. The system was developed on a Lenovo T450S laptop with Windows 10 Pro and an Intel(R) Core (TM) i7-5600U CPU up to 3.2 GHz and 12 GB RAM. The laptop also hosted the SQL-Server instance within the LAN.

## Results

### The windows client

The Windows client is the working environment for the scientists and divided into the four sections *Data View*, *Plot View*, *Lab Log* and *Database View*.

**Fig. 2:**
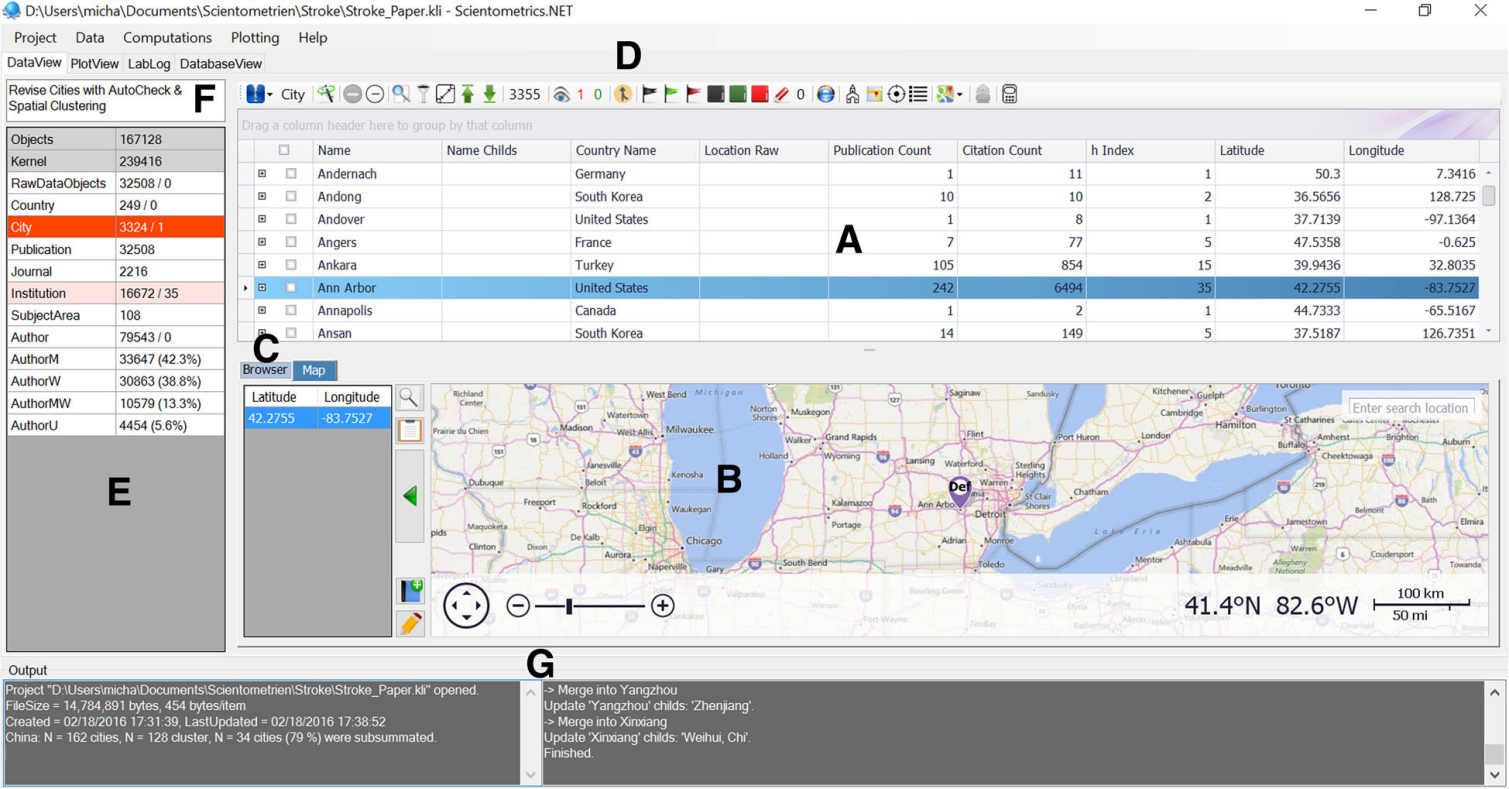
The Data View. The integration, inspection and analysis of data take place within the Data View. The center data table (**a**) displays the selected object data (here: city data). Its corresponding geographic location is indicated in the map below (**b**). An integrated web browser (**c**) supports the integration process by generating search queries, e.g. regarding the gender of a given forename. The buttons above the data table (**d**) enable the functionality to exclude, include, mark, view, manipulate (e.g. merge) and analyze selected objects. On the left side the different (selectable) object types and their numbers are shown (**e**). The number after the slash ('/') represents the count of items to be checked by the user. Above this panel a process-coordinating to-do-list is given to support the user during the different analyzing steps (**f**). The list is automatically updated as a function of the current process state. Notifications and status messages are displayed in two output windows at the bottom (**g**)

**Fig. 3:**
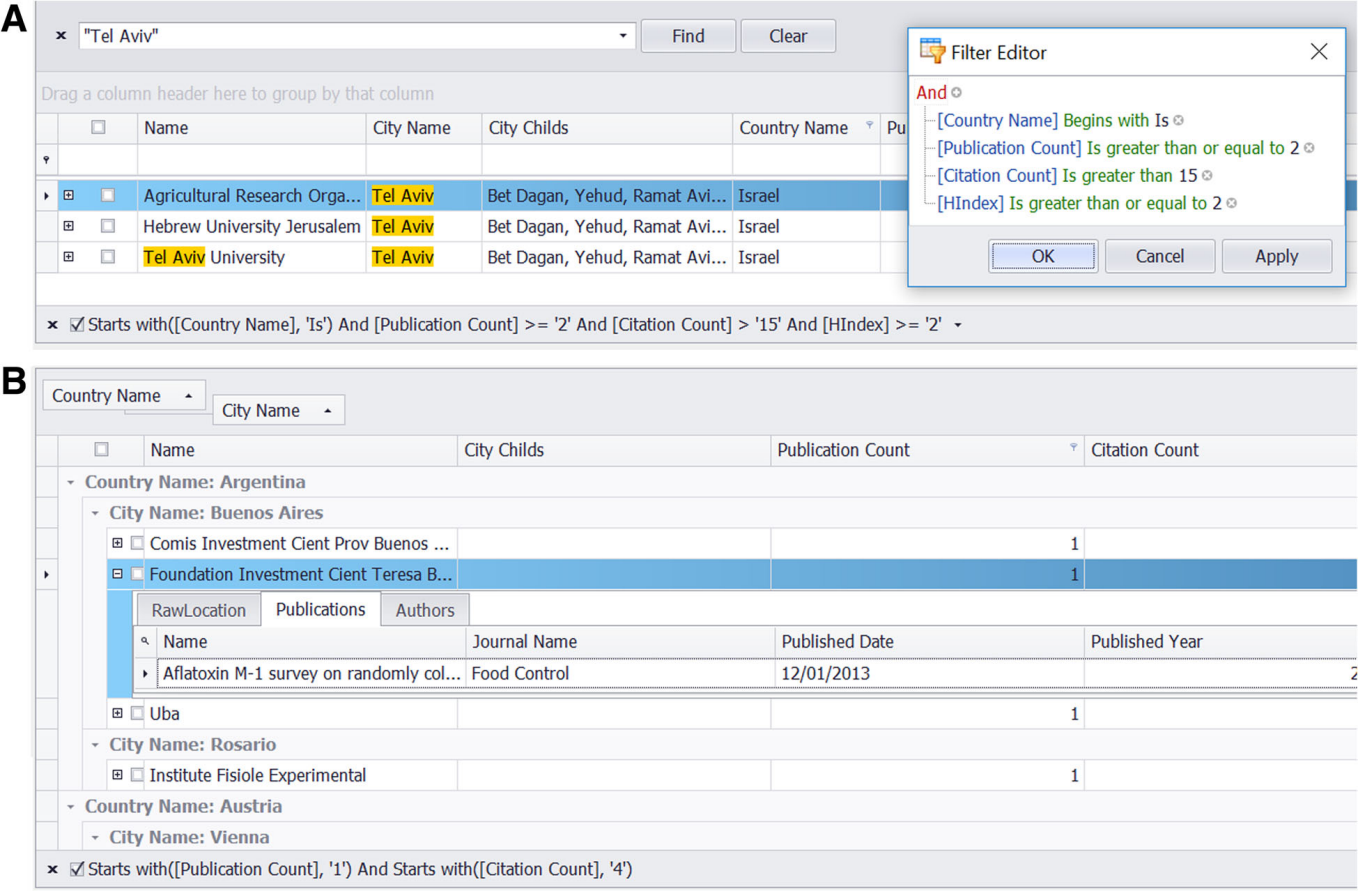
Elementary data table functions. **a** Searching & Filtering: The data table supports the finding and filtering of data by applying a free text search. Here a whole word search for the word “Tel Aviv” results in four hits. Additionally, a complex filter mechanism (“Filter Editor”, on the right) allows the arbitrary selection of data rows by their specific column values. **b** Grouping & Master-Detail-Tables: The data table allows the grouping of data against multiple columns. This fundamental operation is used to organize data rows into a tree which facilitates the inspection of data. In this example the institutions are grouped firstly by country names, and secondly, by city names. Finally, providing a master-detail table technology allows the elegant inspection of object associated data. Here, all publications are listed that are associated to a selected institution

The integration, inspection and analysis of data take place within the Data View. As illustrated in Fig. 2, the center data table displays the selected object data enabling a variety of view, search and filter options, in particular a free text search, a complex combinatorial filter, a master-detail table technology and a sophisticated grouping technology which is used to reorganize the data rows into a tree (Fig. 3). Furthermore, the *Data View* brings together a wide range of functionality to exclude, include, mark, manipulate (e.g. merge) and analyze selected objects. It should be noted that during the analysis process it is possible to exclude and include selected objects at any time; the statistical evaluation is updated accordingly.

According to the strategic focus on geographic information the corresponding geographic location of each localizable object is indicated in a map control below. The map control – based on either BingMaps or OpenStreetMaps offering a classical map view, a satellite view or the combination of both – allows the intuitive inspection of regions at different zoom levels, the localization of cities, the read out of geo-coordinates and the (re)definition of cities. An integrated web browser supports the integration process by generating automatic search queries, e.g. regarding the gender of a given forename or the portrait of a selected author. A process-coordinating to-do-list is given to support the user during the consecutive analyzing steps. This list, which defines a standard operating procedure, is automatically updated as a function of the current process status.

**Fig. 4:**
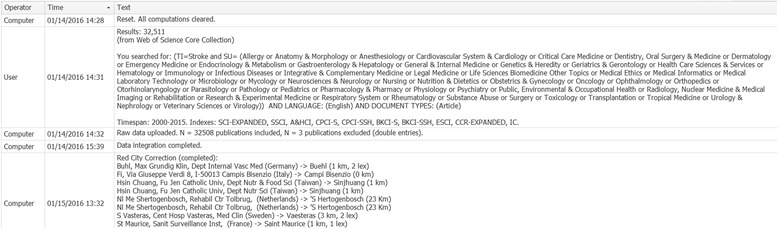
Lab Log. The *Lab Log* allows the management of a project-specific logbook. For the sake of clarity, an operator and a timestamp label each entry. In addition to user entries, the computer system automatically adds relevant project information

The *LabLog* allows the management of a project-specific lab book (Fig. 4). It serves as an organizational, documentation and memory tool for parameters, hypothesis and the interpretation of results and can also have a role in protecting any intellectual property that comes from the user. In addition to user entries, the system automatically adds relevant project information, e.g. the results of an algorithmic process. For the sake of clarity, each entry is labeled by an operator name and a timestamp.

**Fig. 5:**
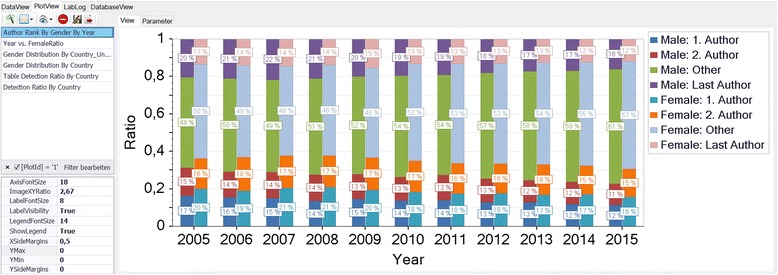
The Plot View. The results of the statistical analysis are illustrated by canonical data plots given in the list on the left. Chart parameters are specified by the property control on the bottom left of the page. Here, the gender-specific distribution of the author’s ranks is bar-plotted as a function of the publication year (left bars: male, right bars: female). Both, figures and underlying data can be exported for further external processing

The results of the statistical evaluation are illustrated by canonical data plots within the *Plot View* (Fig. 5). Predefined chart parameters like font sizes or legend visibility are adjustable for fine-tuning making the chart ready for publication. In addition, figures and data can be exported for further processing. Finally, the password protected *Database View* offers the main functions for maintaining the database.

### Context-sensitive dictionaries

By default, the integration of raw bibliometric data is done by identifying predefined, database-located data objects like city or country names in the particular raw data string. In order to improve the quality features of integration, essentially its adaptively, robustness and error tolerance, a set of dictionaries was introduced for the entity type’s city, country, journal and institution. Mathematically, a dictionary is a collection of key and value pairs in the sense of a partial function. Concerning cities and institutions this classic dictionaries were extended by context-sensitivity, i.e. the key-value-pair is bounded to a particular context, e.g. to a country or a city, respectively. By that, e.g. the city substitution is country-specific.

**Fig. 6:**
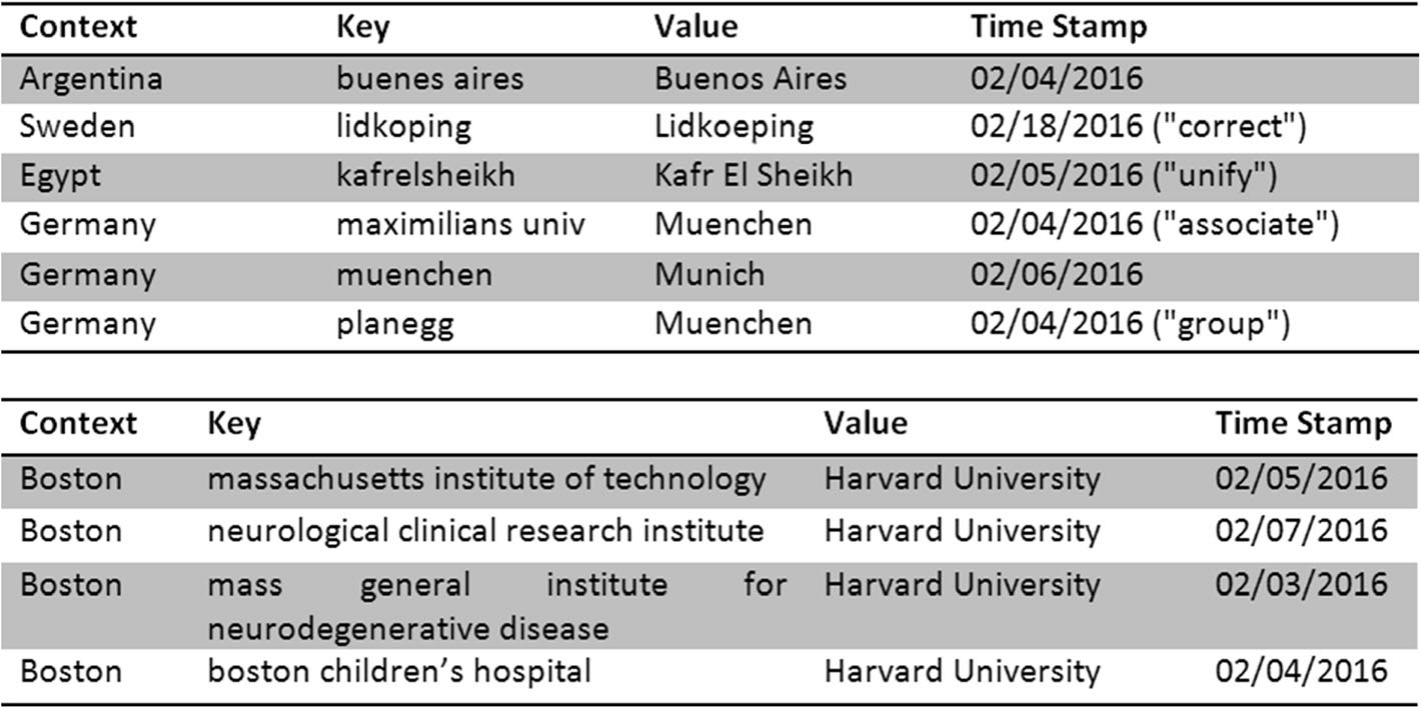
A context-sensitive dictionary for learning the integration & grouping process. (*Top*) Structure and main functions exemplified by the dictionary of cities. Given a particular context, a key (consisting of only lowercase letters and spaces) is associated with a value in the sense of a partial function (key-value-pair). In the case of the city dictionary the context is given by the country of the city. By applying the dictionary during the data integration process corrections of incorrectly written (“correct”) or versatile names (“unify”) can be made. Furthermore, dictionaries allow learning associations across different data types improving the identification rate significantly. In this example the institution name ‘maximilians univ’ is correctly maped to the corresponding city name ‘Muenchen’ (“associate”). Finally, the city dictionary allows the subsuming of nearby cities (“group”). In this example the suburb ‘planegg’ is subsumed under the term ‘Muenchen’. Substitutions once learned can by expanded by function compositions. Here, through the intermediate step of ‘Muenchen’, ‘planegg’ is gradually replaced by ‘Munich’. Recursions are avoided by prohibiting cycles. For easy reproduction and correction, each entry within the dictionaries is provided with a time stamp. (*Bottom*) Grouping exemplified by the dictionary of institutions. Dictionaries may be used to group data. Thus, e.g. given a common context (here: a city name) an institution can be considered as representative for many other institutions. Here the given institutions with the common context “Boston” are subsumed under the term “Harvard University”. Generally, such dictionaries exist for cities, countries, journals and institutions. As a basic principle, each reclassification of a given entity during the working process can be stored by updating the corresponding dictionary, respectively. The consistent use of the learning function allows the complete algorithmic reintegration of formerly integrated data

**Fig. 7:**
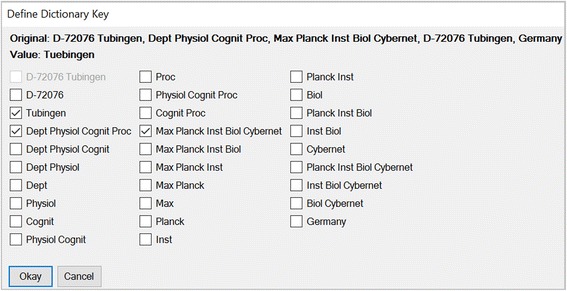
Definition of new dictionary entries by identifying optimal keys. As a basic principle, each (re)classification of a given entity during the working process can be stored by updating the corresponding dictionary. In this example the incorrectly spelled city name *Tubingen* was corrected with *Tuebingen* (Value, second bold line). To store this substitution the user has the option to select valid keys defining new Key-Value-Pairs in the corresponding dictionary. In case of the city dictionary not only the original fault value is given, but rather a list of keys created by the combinatorial processing of the raw location string (first bold line). A key is valid, when its distinct mapping to the particular key is correct. An optimal key is a valid key which is not substitutable by another valid key. Here, the keys *Tubingen* (=original fault value), *Dept Physiol Cognit Proc* and *Max Planck Inst Biol Cybernet* (both institutions are unique in Germany) are optimal. All other keys are not valid due to their ambiguity (e.g. *Max Planck*) or not optimal to their substitutability (e.g. *D-72076 Tubingen*). To simplify the distinction between valid and optimal keys the program checks for key redundancy immediately after (de)selecting a key. In this example the valid key *D-72076 Tubingen* is inactivated due to its substitutability by the activated key *Tubingen*. Apparently, the role of the user is to identify optimal keys

By applying the dictionaries during the integration process corrections of incorrectly written (“correct”) or versatile names (“unify”) can be made. Furthermore, it is possible to learn associations across different data types improving the identification rate – e.g. in the case of a wrong spelled city name – significantly. In the example in Fig. 6a the institution name ‘maximilians univ’ is correctly mapped to the corresponding city name ‘Muenchen’ (“associate”). Moreover, dictionaries are used to group cities and institutions that can be considered as representative for others (normalization). For example, in Fig. 6a the suburb ‘planegg’ is subsumed under the term ‘Muenchen’. Another example is shown in Fig. 6b, where the given institutions with the common context “Boston” are subsumed under the term “Harvard University”. Substitutions once learned are expandable by function composition; recursions are avoided by prohibiting cycles. As a basic principle, each (re)classification of a given entity during the working process can be stored by updating the corresponding dictionary, respectively. In particular, the user has to define new dictionary entries by identifying optimal keys (Fig. 7). As a consequence, once learned data integrations and modifications can be reconstructed almost completely. Each entry within the dictionaries is provided with a time stamp for easy maintenance.

### City integration

The most difficult and computationally expensive part of the integration process is the correct identification of the city with a publication. Specifically, given the country, the objective is to determine reliably the correct city name from a raw location string like “Wageningen Univ, Div Human Nutr, NL-6700 EV Wageningen, Netherlands” despite variant or wrong spellings. This step is essentially due to the need of retrieving the corresponding city geo-coordinate for further data processing, e.g. the revision of identical, but variant spelled authors.

To accomplish this task, a lexical (whole-word) search routine tries to identify both, predefined city names and, preferred, keys of the city dictionary in the raw string. The preference of dictionary keys stems from the fact that dictionary keys are per definition the result of a user supervised process and hence more meaningful than predefined city names. Cities and corresponding geo-coordinates are predefined in the SQL-database.

This form of city identification causes two main problems:

1. The existence of non-identifiable cities caused by a wrong or variant spelling. Under the assumption of using an unlearned data base approximately 5–10 % of all locations fall into this category. These cities are assigned to a red status (“red cities”). It is important to note, that the use of a learning dictionary reduces this problem after few integration rounds significantly.
2. The existence of identified, but ambiguous city names which can be caused, e.g. by the non-consideration of additional information given in the raw location string, e.g. the specification of a federal state or a zip code. Such cities are assigned to a green status (“green cities”).

In both cases a user-supervised post processing is required. Its various steps are described in the following.

### Integration of red cities

**Fig. 8:**
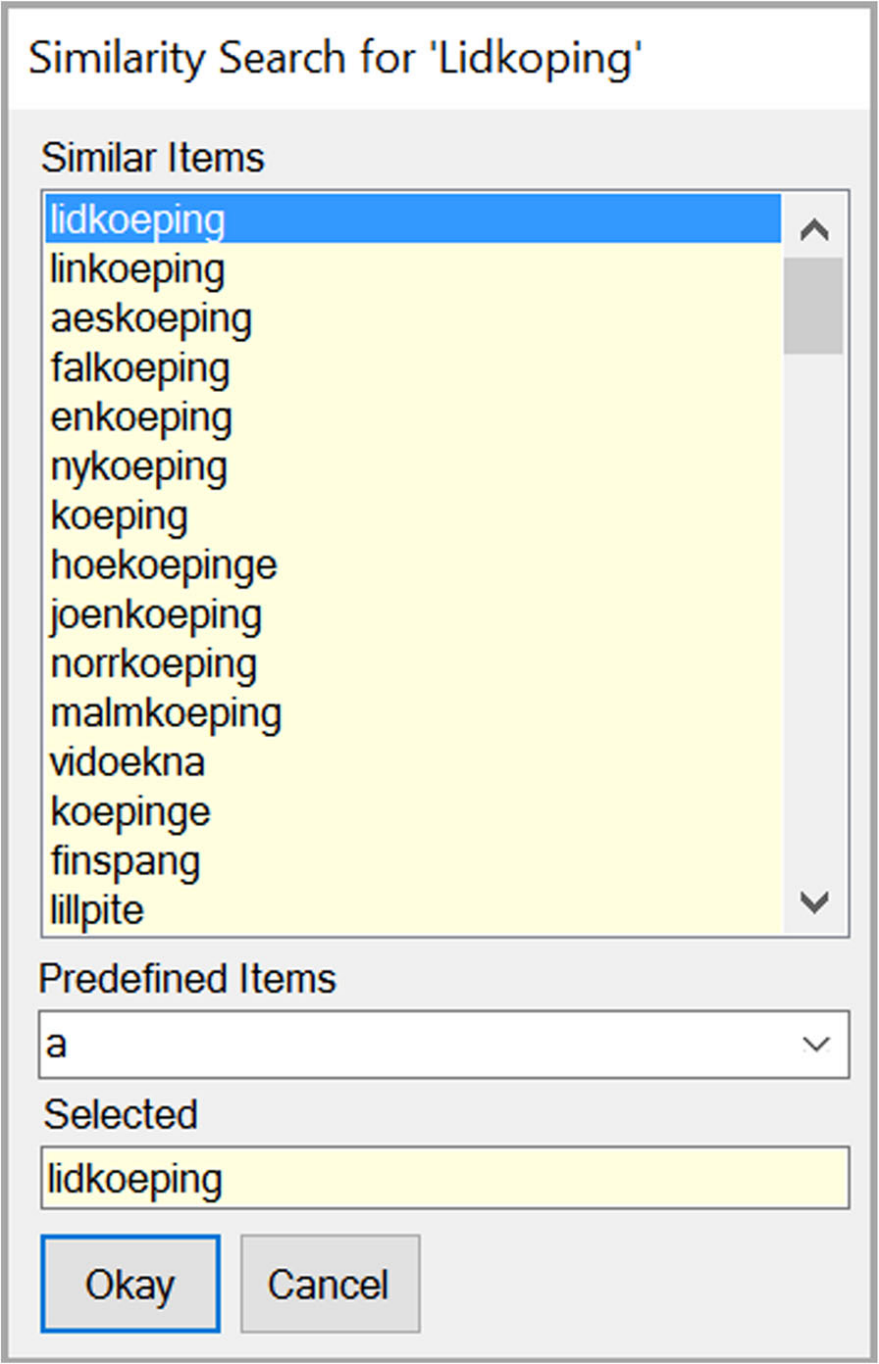
Correcting cities by Similarity Search. In order to correct a wrong or variant spelling (here *Linkopin* instead of *Linko* ***e*** *pin*, Sweden) a rapid lexical similarity analyzer (based on the Levenshtein distance) suggested the most similar predefined object names
(iii) Finally, the integrated web browser enables the user to generate automatic queries to Google Maps. In case of a positive identification the geo-coordinates of the city – automatically extracted from the address line of the browser – are used to make predefined cities within a given distance (typically *r* = 20 km) available for selection. In this way, the integration of red cities can be achieved reliably.

(i) To integrate cities with a red status it is recommended to first apply the BingMapSearch-Service (SOAP-Search-API, Microsoft). Specifically, starting from a given location string, multiple combinatorial queries are generated and executed. In case of a positive identification the identified city name and its geo-coordinates are returned. On the basis of this information, predefined cities located inside a particular radius around the target (typically *r* = 20 km) are provided and ordered by their lexical similarity (Levenshtein distance) to the target. This process iterates automatically through all red (= unidentified) cities. After this operation the user has the option to select between the suggested cities. If no corresponding predefined city equivalent exists, the user can define a new city. By this procedure about 40 % to 50 % of the previously unidentified cities are resolved.
(ii) In order to correct a wrong or variant spelling (e.g. “Linkopin” instead of “Linkoepin”, Sweden) a rapid lexical similarity analyzer (based on the Levenshtein distance) suggested the most similar predefined city names for selection as illustrated in Fig. 8.

It is important to note, that in all three cases the city dictionary is updated accordingly.

### Integration of green cities

The definitive assignment of the green cities (= identified, but ambiguous city names) is similar to the identification of the red cities. Again multiple combinatorial queries – including additional local information like the federal state – are sent to the BingMapSearch-Provider. In case of a positive identification (in about 80 % of the cases) the geographically nearest city is selected from the list of possible (i.e. equally named) cities. If the chosen city is outside a defined maximum distance (typically d = 20 km) the user can define a new city. In order to enable the system to learn this decision, the selection is stored automatically in the database for subsequent city integrations.

This latter procedure is legitimated by the paradigm that in common only one city contributes significantly to scientific articles within a set of identical city names in a country (e.g. Berlin, capital town of Germany and Berlin, a small village in Schleswig-Holstein). If this rule is violated (e.g. Arlington in Virginia and Arlington in Illinois) the related federal state, zip code or any other additional local information has to be manually incorporated in the city name.

### Spatial clustering of cities

Depending on the scientific question posed, it is recommended to perform a spatial clustering of all cities after the integration process. This operation is realized by implementing the DBSCAN (= Density-Based Spatial Clustering of Applications with Noise) algorithm [12], a density based cluster algorithm which can find arbitrarily shaped clusters in the data (e.g. a city region along a river) or clusters that are completely surrounded by a different cluster (e.g. exurbs). As a parameter the user has to define the maximum distance ε which specifies how close points should be to each other to be considered a part of a cluster (typically ε = 50 km). In this way city regions are formed, whereby the city with the highest publication count defines the name of the region whereas remaining cities are subsumed as city child’s (e.g. city region ‘Boston’ with child’s ‘Cambridge’).

As a result, the scientific relevance of a versatile and partitioned region is more adequately captured than using a pure city-based approach often underestimating the scientific impact.

### Gender identification

The gender of an author is algorithmically determined by its forenames und differentiates between four different groups (male, female, unisex and undefined). The analysis is context-free, i.e. solely based on the forenames of the author, additional information like the related countries are not included. Conceptually, each gender of a forename is determined on the basis of data table which currently contains about 76,600 forenames defining the genders as *male*, *female* and *unisex*. The number of male and female names is approximately equal (N_male_ = 30,544, N_female_ = 30,874, N_unisex_ = 15,149). In the case of multiple forenames for each forename the gender is determined. Subsequently the gender is considered as male (female) if more male (female) forenames exist. Otherwise it is marked as unisex. Undefined authors can be revised by applying a second algorithm which requests predefined webpages specialized in determining the gender by first names. Both algorithms are fully automated, thus no further user interaction is needed. Finally, the user can revise unisex and undefined authors manually by applying the integrated web browser, e.g. to generate automatic queries to Namepedia (www.namepedia.org) or to identify the author by a simple name search. In all cases the gender table is updated accordingly.

**Fig. 9:**
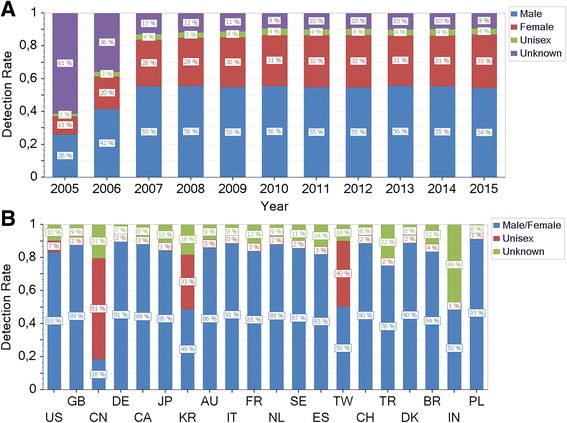
**a**: Gender detection rate by year. The algorithm gender determination typically succeeds reliably for articles that are published since 2007 as indicated by this example with relative constant percentages of male (55 %), female (31 %), unisex (4 %) and unknown (11 %) gender, respectively. Before 2007 the dominance of initials prevents the correct lexical gender identification by first names. **b**: Country-specific gender detection rate. The country-specific distribution of the algorithmic gender detection documents a clear dependence on the authors’ country with high detection rates (>80 % male or female) for the majority of the top 20 productive countries and low detection rates for the Asian states China (CN), South Korea (KR) and Taiwan (TW) that are characterized by a high rate of unisex names and India (IN) with almost 50 % of unknown names

**Fig. 10:**
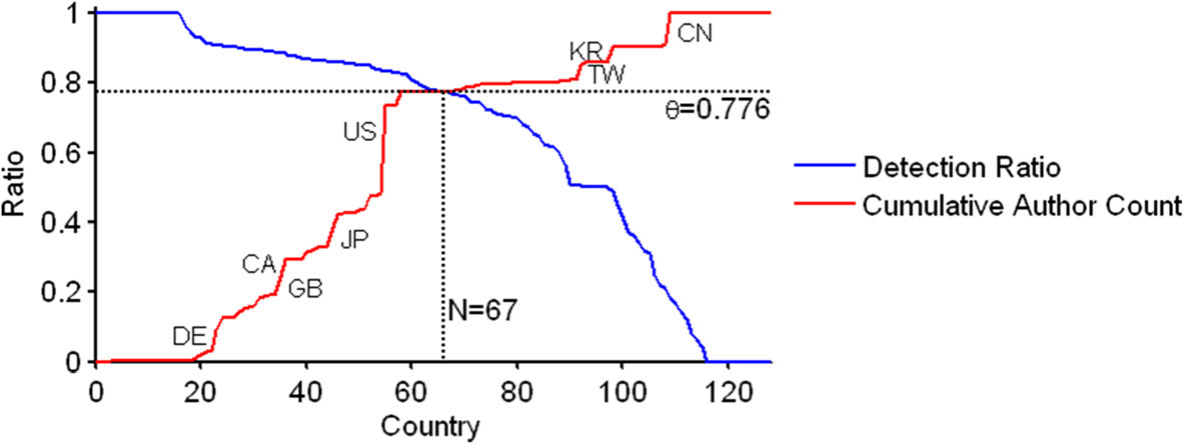
ROC-Curve for algorithmic gender detection. The intersection point of the country-specific gender detection ratio (sorted in descending order, blue line) and the corresponding cumulative author count (red line, countries with a large amount of authors are indicated by an additional label) defines a adaptive threshold detection criterion θ for the inclusion of a country in the analysis. By definition, θ also defines the fraction of considered authors. In this example countries with a detection rate of at least theta = 0.776 (i.e. 77.6 % of all authors from *N* = 67 countries) are included in the analysis

It is worth to note that the algorithmic detection succeeds reliably for articles that are published after 2006. By contrast, the dominance of initials prevents the correct gender identification in older articles (Fig. 9a). The quality of algorithmic gender detection depends crucially on the authors’ country as illustrated by Fig. 9b. Specifically, the country-specific distribution of the algorithmic gender detection documents high detection rates (>80 % male or female) for the majority of the top 20 productive countries with the exception of the Asian states China (CN), South Korea (KR) and Taiwan (TW) that are characterized by a high rate of unisex names and India (IN) with almost 50 % of unknown names. By constructing a ROC curve the software determines automatically a natural threshold criterion for the inclusion of a country into the statistical evaluation, see Fig. 10.

### Process plan

**Fig. 11:**
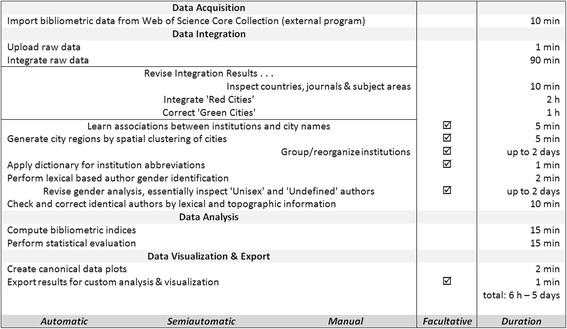
Process plan to perform a scientometric analysis by Gendermetrics.NET. The process is divided into the four sections ‘Data Acquisition’, ‘Data Integration’, ‘Data Analysis’ and ‘Data Visualization & Export’. According to their degree of automation, the program steps are further classified as ‘Automatic’ (= complete algorithmic realization without direct supervision, autonomous algorithms), ‘Semiautomatic’ (= partial algorithmic realization with supervision, supervised algorithms) and ‘Manual’ (= user decisions without algorithmic support) (left column). Optional program points are marked by a checkbox (middle column). Assuming a fairly typical project size of *N* = 25.000 publications, an estimated processing time is specified for each program point as well as for the whole analysis (right column). Please note a rapid integration mode on the topographical level of countries instead of cities is provided, increasing the integration speed by a factor of 50

The following steps outline exemplarily a complete bibliometric analysis using Gendermetrics.NET (Fig. 11). To provide a better overview, the analysis process is divided into the four sections data acquisition, data integration, data analysis and data visualization & export. According to their degree of automation, the program steps are further classified as ‘automatic‘ (= complete algorithmic realization without direct supervision), as ‘semi-automatic‘ (= partial algorithmic realization with supervision) or as ‘manual‘ (= user decisions without algorithmic support). Furthermore, to give quantitative guide values, e.g. an estimated average processing time T per step, an average project size of *N* = 25,000 articles is assumed. In common practice, after each step a manually named safety copy of the project is done.

The first step within the analysis is the data acquisition which is characterized by the read out of raw bibliometric data from the Web of Science Core Collection (T = 10 min). The data is imported as a tab-delimited UTF-8 (=8-Bit Universal Character Set Transformation Format) formatted text file. Since the number of entries is limited to a maximum of 500 entries per download the whole data is distributed over several files (here *N* = 50).

After data acquisition the data integration process starts with uploading the raw data into a (not necessarily new) Gendermetrics.NET project (T = 1 min). In this step, the data is reunified within the project and corrected for double entries that are characterized by identical titles, authors, publication dates and journals. The subsequently automatic integration process (T = 90 min, approximately 5–10 items/s) can be interrupted at any time, saved and continued later, as required. Furthermore, due to the multi-user design different integration processes can be performed simultaneously. During the integration process an estimated finish time is given. Additionally, a rapid integration mode on the topographical level of countries instead of cities is provided, increasing the integration speed by a factor of 50.

After the successful process of integration, the results have to be revised by the user. According to their natural dependencies (e.g. a city is identified in a country) the revision order is: Countries, cities and institutions.

Normally, countries, journals and subject areas are integrated flawlessly, in rare cases an unidentified country has to been corrected manually (T = 10 min). In the next revising step, the semi-automatic integration of both, unidentified (red) and ambiguous (green) cities is done supported by diverse algorithms as documented above (T = 2 h, resp. 1 h).

After their correct integration the user has the option to store the associations between institutions and cities (T = 5 min). This step is done before both, substituting the institution abbreviations for better reading and reorganization of the institutions (up to 2 days) with the omission of institution names. To create city regions a spatial clustering of the cities can be performed, optionally (T = 5 min). In the next step a lexical based author gender analysis is performed.

Subsequently, the user has the option to revise the classification of the ‘unisex’ and ‘undefined’ authors by applying web services. The subsequent lexical and topographic check for identical authors marks the last step of the integration process. Here, authors who might be identical (i.e. same name, different or similar first names, different cities) are marked for inspection; authors who are most likely identical (i.e. same name, similar first names and shared cities) are merged automatically. Similar first names are thereby defined as potentially equal by substring containment, e.g. ‘Mark A’, ‘M A’ and ‘Antonio M’.

The following analyze process is characterized by the computation of the bibliometric indices (like publication count, citation-count, topic-specific h-index and topic-specific productivity index) for each object type and the subsequent statistical evaluation including a variety of gender-related computations which can be done with respect to institutions, cities, city regions, countries, subject-areas and journals. One key element is the combined analysis of both, relative frequency and chances of female authors to be a author at a given authorship rank. Finally, all results are illustrated by canonical data plots and are exportable for further analysis and visualization.

## Discussion

The software suite Gendermetrics.NET is a simultaneous multi-user tool to allow seamless integration, inspection, modification, evaluation and visualization of bibliometric data. It extends classical approaches by offering gender and location related analysis tools. By providing an adaptive and almost fully automatic integration and analyze process, the inter-individual variability of analysis is kept at a low level. Gendermetrics.NET is intended for scientists who are interested in bibliometric analysis with a special emphasis on a topographical gender analysis. Depending on the scientific question, it enables the user to perform a scientometric analysis including its visualization within a short period of time.

The server-client architecture reflects the idea of data organization which is characterized by the separation of global and project-specific data. Decentralized (client-side) updates of the database are used to improve subsequent integration, resp. classification processes in the sense of a global learning function. By default, a simultaneous multi-user mode is supported as well as a stand-alone mode after successful data integration.

One key paradigm of analysis is the consideration of the geo-coordinates of each involved city. By this feature, various topographical analyses that are beyond the mere country, state or city level can be realized. Furthermore, the system offers the option to cluster cities spatially by their coordinates using a fast, robust and unsupervised algorithm (DBSCAN, section). This operation results in city regions representing a new spatial level between cities and states, resp. countries. The cluster algorithm can be used to overcome the local fragmentation of a city region which can result in an underestimation of its scientific impact in a solely city-based approach.

Algorithmically, the most challenging part of integration is the correct identification of the city (including its geo-coordinates) as mentioned above. Various methods including both, offline (database-based) and online (web search-based) methods are provided. Among the advantages of offline (database-supported) data integration there are a fast mode of operation and the possibility to update the underlying data in terms of a learning function. The major drawback is the incompleteness of data. By contrast, the major advantage of online (web-based) data integration is the access to large amounts of continuously updated and more comprehensive data. Its main disadvantages are the relative slowness of the query process and the missing capability to update the underlying database individually. Moreover, only a limited number of queries are free of charge (currently the BingMapsSearch-Provider has a limit of 50,000 queries/day, basic & not-for-profit key). For this reason the online-based identification is exclusively made for unidentifiable cities.

By construction, the database-supported city integration is context-free, i.e. additional location-specific information is not considered. For example, the raw location string “Univ Texas Arlington, Dept Bioengn, Joint Grad Program, Arlington, TX 76019 USA” refers unambiguously to Arlington in the U.S. state Texas and not to e.g. Arlington in Virginia. The reason for this critical restriction, which requires at least one further post-processing step, is the enormous complexity required to build an appropriate database. However, the capability of the system to learn the association between institution and city reduces this problem significantly.

Another disadvantage regarding the algorithmic city integration is the usage of the older BingMaps-search version (SOAP-Search-API). In contrast to the currently latest version (REST-API, used in BingMaps 2016) this search API shows a significantly lower hit rate. Unfortunately, the newer REST-API has not yet been integrated in the DevExpress library (personal communication). This will be achieved hereafter.

Apart from the usual bibliometric indices (citation & publication count, h- and productivity-index, author’s ranking) [13] and classical collaboration analysis [4], the statistical evaluation of the data includes a variety of gender-related computations which can be done institution-, city-, city region-, country-, subject-area- and journal-specific. The gender of an author is thereby determined by its forenames und differentiates between four different groups (male, female, unisex and undefined). An unisex (epicene) name is a given name that can be used regardless of the person’s sex. Frequently, names have gender connotations that differ from country to country (e.g. ‘Andrea’ in Italy an exclusively male forename, whereas in Germany used only for female persons). Despite that, the algorithmic sex determination of an author is context-free, i.e. without including additional information like the related countries. One reason for that is that due to international mobility it is not quite possible to conclude from the related country to the nationality of the author, particularly since some authors are associated to institutions of different countries.

By manual reconsideration, e.g. by using the integrated web browser and automatically generated queries (e.g. search for an image to determine precisely the author’s gender), a subsequent reclassification of ‘unisex’ or ‘undefined’ authors is possible. However, despite various technical tools this procedure is expensive and depends critically on the preciseness and persistence of the evaluator introducing an undesirable inter-individual variability into the analysis. Moreover, the success of identification depends directly on the Internet presence of the particular author (is there a photo available?). But even if a careful and complete correction is done, due to a lack of information (in about 80 % of undefined authors the first name consists of initials) or the usage of gender-neutral forenames – especially in Asian countries, the gender of many authors remains unclear. For this reason, it is plausible to skip the manual post-processing in favor of a fast (<2 min), fully algorithmic and therefore reproductive detection. For articles later than 2008 the high algorithmic detection rate of about 80 % enables the investigator to make valid propositions about the underlying population using the methods of statistical inference, particularly in comparision to previously published studies with significant lower detection rates [10].

The extension of the gender-database will result in even higher identification rates. In future, this situation may be tackled more efficiently by using author identification numbers that are associated with additional personal information (e.g. ORCID, UIAN) [6].

## Conclusion

In summary, a new software suite for analyzing gender representations in scientific articles was established. The system is suitable for the comparative analysis of scientific structures on the level of continents, countries, cities, city regions, institutions, research fields and journals.
